# Green Synthesis of Co-Zn Spinel Ferrite Nanoparticles: Magnetic and Intrinsic Antimicrobial Properties

**DOI:** 10.3390/ma13215014

**Published:** 2020-11-06

**Authors:** Alexander Omelyanchik, Kateryna Levada, Stanislav Pshenichnikov, Maryam Abdolrahim, Miran Baricic, Anastasiya Kapitunova, Alima Galieva, Stanislav Sukhikh, Lidiia Astakhova, Sergey Antipov, Bruno Fabiano, Davide Peddis, Valeria Rodionova

**Affiliations:** 1 Institute of Physics, Mathematics and Information Technology, Immanuel Kant Baltic Federal University, 236041 Kaliningrad, Russia; asomelyanchik@kantiana.ru (A.O.); elevada@kantiana.ru (K.L.); spshenichnikov1@kantiana.ru (S.P.); 2 Institute of Structure of Matter–CNR, 00016, Monterotondo Stazione, 00015 Rome, Italy; mabdolrahimi@os.uniroma3.it; 3 Department of Chemistry and Industrial Chemistry (DCIC), University of Genova, 16146 Genova, Italy; S4193366@studenti.unige.it; 4 School of Life Science Immanuel Kant Baltic Federal University, 236041 Kaliningrad, Russia; aikapitunova@stud.kantiana.ru (A.K.); agalieva@stud.kantiana.ru (A.G.); ssukhikh@kantiana.ru (S.S.); lastakhova@kantiana.ru (L.A.); santipov@kantiana.ru (S.A.); 5 Research Center “Molecular basis of Biotechnology of Living Systems”, K.G. Razumovsky Moscow State University of Technologies and Management (the First Cossack University), 109004 Moscow, Russia; 6 Department of Civil, Chemical and Environmental Engineering, Polytechnic School, University of Genova, Via Opera Pia 15, 16145 Genova, Italy; brown@unige.it

**Keywords:** magnetic nanoparticles, cobalt ferrite, zinc ferrite, magnetic properties, antimicrobial

## Abstract

Spinel ferrite magnetic nanoparticles have attracted considerable attention because of their high and flexible magnetic properties and biocompatibility. In this work, a set of magnetic nanoparticles of cobalt ferrite doped with zinc was synthesized via the eco-friendly sol-gel auto-combustion method. Obtained particles displayed a room-temperature ferromagnetic behavior with tuned by chemical composition values of saturation magnetization and coercivity. The maximal values of saturation magnetization ~74 Am^2^/kg were found in cobalt ferrite nanoparticles with a 15–35% molar fraction of cobalt replaced by zinc ions. At the same time, the coercivity exhibited a gradually diminishing trend from ~140 to ~5 mT whereas the concentration of zinc was increased from 0 to 100%. Consequently, nanoparticles produced by the proposed method possess highly adjustable magnetic properties to satisfy the requirement of a wide range of possible applications. Further prepared nanoparticles were tested with bacterial culture to display the influence of chemical composition and magnetic structure on nanoparticles-bacterial cell interaction.

## 1. Introduction

Magnetic spinel ferrites nanoparticles with general chemical formula MFe_2_O_4_ (where M = Co^2+^, Ni^2+^, Mn^2+^, Zn^2+^…) offer great magnetic property tunability by varying their size and chemical composition [1,2,3]. This kind of material is very attractive for biomedical applications such as magnetic resonance imaging (MRI) contrast agents and mediators of heating in magnetic fluid hyperthermia [4,5,6,7,8]. The possibility to control the movement of magnetic nanoparticles (MNPs) by applying a magnetic field gradient opens the doors for targeted drug- [9], genes- [10], or peptides-delivery, for instance, of antimicrobial or antifungal peptides [11]. The use of doped ferrites for antimicrobial or antifungal delivery for water purification is also within the scope of interest because of the possible effect on the dynamics of bacterial growth of some metals ions (silver, zinc, and copper) incorporated with MNPs and, because of the possibility to easily filter them by using a magnetic field [11,12,13]. Additionally, perspectives of the use of magnetic nanoparticles in water purification are reinforced by their effectiveness to absorb heavy metals [14,15,16].

Chemical engineering of spinel ferrites results in the modification of their structural and magnetic properties [17,18,19,20]. Magnetic spinel ferrites consist of two antiferromagnetically ordered sublattices: the tetrahedrally and octahedrally coordinated A- and B-sites. According to Néel’s model [1,21], in ferrimagnetic spinels, the net magnetic moment can be ascribed to the non-equality of magnetic moments of two sublattices (i.e., μ = μ_a_ − μ_b_). The distribution of divalent ions in octahedral sites represents inversion degree γ. Magnetic properties of spinel ferrite nanoparticles (i.e., saturation magnetization M_S_ and coercivity force H_C_), are strongly correlated with both chemical composition and value of inversion degree γ. 

Recently, quite high M_S_ values have been observed in Zn-doped spinel ferrites (i.e., Zn_x_Fe_x_Fe_2_O_4_ [4,22,23,24], Zn_x_Co_1_ − _x_Fe_2_O_4_ [5,25,26,27], Zn_x_Ni_1_ − _x_Fe_2_O_4_ [28], Zn_x_Mn_1_ − _x_Fe_2_O_4_ [4,26] MNPs where M_S_ increase while x (i.e., Zinc content) is less than ~0.5. For example, the Cheon team observed an extremely high M_S_ value of 161 and 175 Am^2^/kg for Zn_0.4_Fe_2.6_O_4_ and Zn_0.4_Mn_0.6_Fe_2_O_4_ respectively [4]. For Zn^2+^_x_Fe^2+^_(1 − x)_Fe^3+^_2_O_4_ ferrites an increase of magnetic moment from 4.0 μ_B_/f.u. (Bohr magnetons per formula units) to 8.3 μ_B_/f.u., according to the (4 + 6x) μ_B_/f.u. rule, was confirmed through density functional numerical calculations [24]. This rule was valid for Zn concentration 0 < x < 0.75 where magnetization reached maximal value and start to decrease. That was explained by the replacement of Fe^3+^ with magnetic moment 5 μ_B_/f.u. in A-site by non-magnetic Zn^2+^ cations. The Co^2+^ in the structure of spinel ferrite makes material magnetically harder keeping the relatively high value of saturation magnetization [29,30,31]. Mameli and colleagues found that the M_S_ at 5 K reaches a value of 157 Am^2^/kg for Zn_0.46_Co_0.54_Fe_2.02_O_4_ synthesized by the high-temperature decomposition method [24]. Barrera et al. synthesized a set of Zn_x_Co_1_ − _x_Fe_2_O_4_ MNPs with x in the range of 0.08−0.56 using a sol-gel auto-combustion method (SGAC) [27]. In their work, authors observed that starting from inverse spinel at x = 0.08 future increase of zinc content to x ~ 0.4 leads to the formation of mixed ferrite where Zn^2+^ forces Co^2+^ migrate to A-site and Fe^3+^ to B-site. It was highlighted that non-equilibrium cation distribution is strongly related to the method of synthesis and, together with spin canting, leads to a non-monotonous change of saturation magnetization. 

Recently several methods were employed in order to obtain Zn and Co co-doped ferrites MNPs such as high-temperature decomposition [5,25], SGAC [27], polyol [32,33], co-precipitation [26,34]. Among them, the SGAC has several advantages: (i) low-cost and eco-friendly reagents; (ii) fast time reaction; (iii) high crystallinity of the particles. The SGAC method utilizing citric acid as a raw material can be considered a green synthesis method [35]. Keeping relatively good magnetic performance of the samples this method indeed does not consume hazardous organic solvents and does not require the use of any specific washing procedure with, for example, acetone as in high-temperature decomposition or polyol methods. Moreover, due to the very high yield of the reaction, no waste excepting nitrogen oxides is present at the end of the process. The applicability in magnetorheological and multiferroic composites of MNPs obtained with this method was discovered [36]. Recently we obtained a set of cobalt ferrite MNPs doped with nickel x = 0, 0.25, 0.5, 0.75 and 1 with gradually changed coercivity and non-monotonically changing saturation magnetization [37]. 

In this work, we used SGAC for the synthesis of cobalt-zinc ferrites MNPs with x in the range 0.15–0.75 to study the evolution of M_S_ and H_C_. The structural and magnetic properties were studied and compared with previously reported work of Ni-doped cobalt ferrite MNPs [37]. Further, we give new insights about the influence of obtained MNPs on bacterial growth in correlation with their magnetic structure. Although the importance of the magnetic structure of spinel MNPs is well investigated in catalysis [38,39], there is a lack of information about its influence on biological activity. Thus, all samples have been investigated using wild-type bacterial *Escherichia coli* (*E.coli*) K-12 MG1655 [40,41]. 

## 2. Materials and Methods 

### 2.1. Nanoparticles Synthesis

Samples of MNPs were prepared by the SGAC method described in detail elsewhere [37,42]. The metal salts Fe(NO_3_)_3_·9H_2_O (Carlo Erba Reagenti SpA, Cornaredo, Italy), Co(NO_3_)_2_·6H_2_O (Scharlab S.L., Barcelona, Spain) and Zn(NO_3_)_2_·6H_2_O (Merck KGaA, Darmstadt, Germany) were used without future purification. The 1-molar aqueous solutions of metal salts in distilled water (DW) were prepared with different a molar ratio (i.e., Zn_x_Co_1-x_Fe_2_O_4_ with x = 0, 0.15, 0.25, 0,35, 0.5, 0.75 and 1). Then, the preliminary prepared 1-molar solution of the same volume of the citric acid (Scharlab S.L., Barcelona, Spain) in DW was added to mixtures of metal salts under magnetic stirring. The pH-level was adjusted to the value of 7 by dropwise adding of 30% ammonia solution (Carlo Erba Reagenti SpA, Cornaredo, Italy). The obtained sol was dried for about 60 min at 150 °C to form a gel; then the temperature was increased up to 300 °C to induce the self-combustion reaction. Obtained powders were collected and grind with an agate mortar (C. Giese Achat-Laborbedarfs, Idar-Oberstein, Germany).

### 2.2. Structural and Magnetic Properties

The X-ray diffraction (XRD) studies were performed with a PW 1830 powder diffractometer (Philips, Eindhoven, The Netherlands) using Co Kα source (λ = 1.78919 Å) in the 2θ geometry in the range of 30–80 degrees. The morphology of the samples was investigated with a JEM-2100 transmission electron microscope (TEM, JEOL, Tokyo, Japan) operating at 30 kV. The magnetic properties were measured with a vibrating sample magnetometer (7400 System, Lake Shore Cryotronics Inc., Weterville, OH, USA) in the field range up to 1.0 T at room temperature (~300 K). The powder samples were fixed with diamagnetic glue in plastic holders to prevent any movement of the powder during the measurements. 

### 2.3. Antimicrobial Activity

All experiments were done using *E.coli* K-12 MG1655 bacterial cells (taken from laboratory collection of IKBFU, Kaliningrad, Russia). First, antimicrobial activity was tested with the disk diffusion method in order to qualitatively define the parameters of the experiment (see Figure S1 and description below). Then quantitative analysis was performed by the optical density (OD_600_) measurement method on a UV/vis spectrophotometer (SmartSpec Plus, Bio-Rad, Hercules, CA, USA) detecting absorbance at 600 nm wavelength every half hour. Bacterial cells were grown aerobically at 37 °C under constant shaking (~120 rpm) in 5 mL of liquid (without adding of agar) hand-made Lysogeny Broth (LB) medium, containing 1% tryptone, 0.5% yeast extract and 1% sodium chloride. In all experimental samples was added 0.5 mg of nanoparticles. To assess the changes in the optical properties of the LB medium after the addition of nanoparticles, its optical density was detected at a wavelength of 600 nm. It was found that the increase of optical density occurred no more than 10% compared with the control. Obtained OD_600_ data values were analyzed using a two-way analysis of variance (ANOVA, GraphPad Prism 7.04 software, Graph Pad Software Inc., San Diego, CA, USA). All OD_600_ data discussed in the Biology section below are statistically significant (*p*-value meanings were ranked by asterisks **** (*p* ≤ 0.0001), *** (*p* ≤ 0.001), ** (*p* ≤ 0.01).

## 3. Results and Discussion

### 3.1. Structural and Morphological Properties

**Figure 1:**
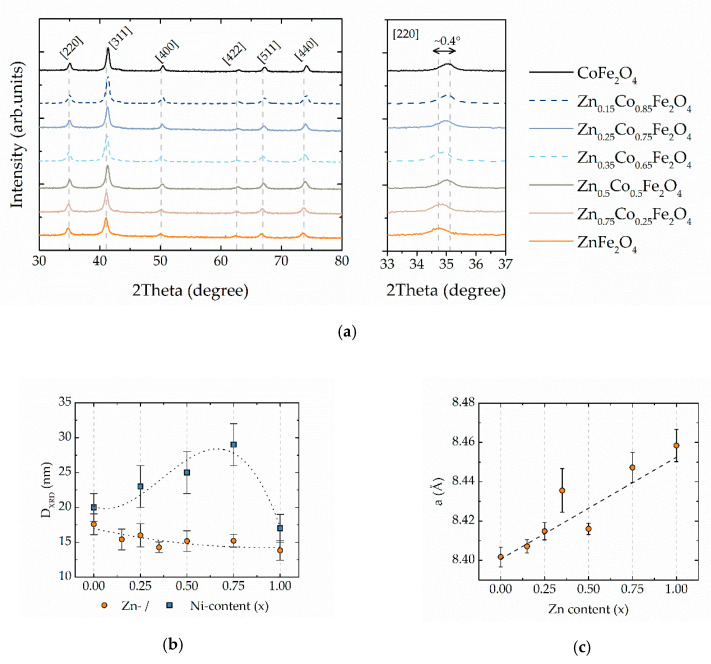
(**a**) diffraction patterns for all samples, in the left panel is a zoomed region for [220] reflection showing the shift of peak position; the Miller indexes for corresponding peaks are reported; (**b**) evolution of grain size (D_XRD_) calculated with Scherrer formula through zinc content compared with the values obtained for Ni-Co ferrite MNPs in ref. [37]. Error bars are the standard deviation of D_XRD_ calculated for 5 most intensive peaks; (**c**) lattice parameter (**a**) as a function of zinc content.

The spinel ferrite structure was confirmed by XRD patterns (Figure 1a) showing for all the samples the presence of spinel cubic structure (card № 00-154-0973 for cobalt ferrite or card № 00-154-0936 for zinc ferrite). Any other phases have been not detected, in contrast to nickel-doped cobalt ferrite MNPs prepared with the same SGAC method where at a high nickel content (x = 0.75 and 1) a small amount of metallic nickel and hematite (α-Fe_2_O_3_) has been observed [37]. The size of crystallites (D_XRD_) was calculated by using the Scherrer formula [43]:

D_XRD_ = K λ/B cos θ, (1)

where K is a crystallite-shape factor (0.94 for spherical particles), B is the full width at the half maximum estimated after fitting of peaks with the Voigt function and θ is the position of corresponding peaks. The D_XRD_ value is slowly decreased over the concentration of zinc (Figure 1b, Table 1). A similar decrease was observed in ref. [27] in zinc-cobalt ferrite MNPs in the range of x = 0.08−0.56. It is worth to underline, that in the case of cobalt-nickel ferrite MNPs obtained with the same method, the smaller size of crystallites was in pure cobalt and nickel ferrite but remarkably higher for mixed Ni-Co ferrite nanoparticles [37,44]. Similarly, the position of main reflections is continuously increasing with the increase of zinc content despite the similar ionic radii of Zn^2+^ (~0.82 Å [45]) and Co^2+^ (~0.82 Å [45]) ions: this can be ascribed to the migration of the smaller Fe^3+^ (~0.67 Å [45]) ions to octahedral sites since zinc ions prefer to occupy the tetrahedral position [25]. The fact that bigger Zn^2+^ occupies the smaller tetrahedral sites is confirmed by the expansion of the lattice parameter a (Figure 1c). The lattice parameter of cubic structure (a) was calculated by the equation $a\;={\;d}_{\mathrm{hkl}}\sqrt{h^{2}+k^{2}+l^{2}}$, where d_hkl_ is the interplanar spacing of a plane with Miller indices h, k and l. The linear dependence of a versus x agrees with Vegard’s law predicting that the lattice parameter of a chemically homogenous mixture will be the approximately weighted mean of two constituents [46].

The TEM-images indicate the presence of non-regular shape of particles with relatively broad distribution (i.e., in the range 10–30 nm) and shape edges confirming the crystalline nature of particles. Examples of typical TEM-images for CoFe_2_O_4_ are presented in the SI (Figure S2).

### 3.2. Chemical Control. of Magnetic Properties

**Figure 2:**
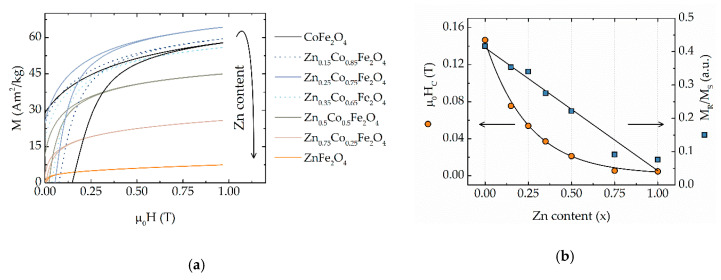
(**a**) The first quarter of M-H hysteresis cycles recorded at 300 K of CoFe_2_O_4_ nanoparticles doped with Zn. The arrow indicates the trend of M_S_; (**b**) Dependence of coercivity H_C_ and reduced remanence M_R_/M_S_ versus Zn-content.

Preliminary magnetic characterization has been performed for all the samples at 300 K. All investigated samples exhibited hysteretic behavior (Figure 2a) with the values of saturation magnetization (M_S_), coercivity field (H_C_) and reduced remanent magnetization (M_R_/M_S_) reported in Table 1. Since almost all M-H curves were not saturated, the Law of Approach to Saturation (LAS) was used to estimate M_S_ values [47,48]:

M = M_S_·(1 − a/H − b/H^2^), (2)

where a and b are free parameters of the fitting. M_S_ first increases with the increase of Zn-content, up to x = 0.25. After this value, M_S_ starts to decrease in good agreement with literature data and may be explained by the cation distribution [5,25,26,27]. The maximal value of M_S_ of ~74 Am^2^/kg was for x = 0.25, which is higher than reported for samples prepared with a similar method, probably because we used a higher reaction temperature, leading to more crystalline particles. For Ni-doped ferrite, we observed maximal value M_S_ of ~69 Am^2^/kg for Ni_0.25_Co_0.75_Fe_2_O_4_ [37]. The coercivity field (H_C_) and reduced remanence (M_R_/M_S_) are decreasing with the increase of zinc concentration (Figure 2b).

**Figure 3:**
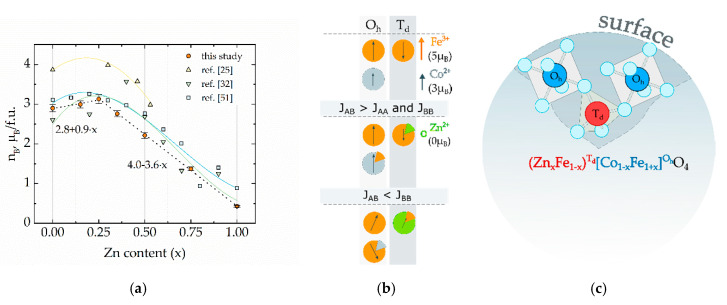
(**a**) Dependence of net magnetic moment per formula unit for cobalt ferrite nanoparticles doped with zinc from this research compared with reference systems [25,32,51]; (**b**) diagram of the evolution of the magnetic structure of cobalt ferrite when is doped with zinc; (**c**) illustration of spinel structure of co-doped Zn-Co ferrite: in nanoferrites the octahedral (O_h_) positions of spinel preferably allocated on the surface rather than tetrahedral (T_d_) positions.

M_S_ values calculated with Equation (3) were converted into the magnetic moment per formula unit in Bohr magnetons to better understand processes associated with filling sublattices in the structure of ferrites. The following equation was used to do that converting [27,45]:

n_B_ = M_S_·M_w_/N_A_·μ_B_, (3)

where M_w_ is the molecular weight, N_A_ is Avogadro constant (6.022 × 10^23^ mol^−1^) and μ_B_ is Bohr magneton (9.274 × 10^−24^ J/T). Figure 3a shows the values of M_S_ in μ_B_ units extracted from M-H curves recorded at 300 K. In the region of 0 < x < 0.25 increase of magnetization is due to a partial migration of Fe^3+^ cations to B-sites. In fact, for low zinc concentration, Zn^2+^ push Fe^3+^ from A to B sites (Figure 3b). If exchange interactions between two different sites (exchange integral J_AB_) are stronger than intra-lattice interactions (J_AA_ and J_BB_), the net magnetization may be expressed as (3 + 7x) × μ_B_, because one uncompensated magnetic moment of Co^2+^ (3μ_B_) is replaced by two uncompensated Fe^3+^ (5 μ_B_) in the octahedral position [4,49,50]. With an increase of concentration of zinc, A-B interaction is weakening. In an extremal case x = 1, J_AB_ and J_AA_ are equal to 0 because the tetrahedral positions are filled with non-magnetic Zn^2+^ ions and B-B interaction leads to the establishment of antiferromagnetic ordering among magnetic moment in the octahedrally coordinated lattice. On basis of this simple model, a general rule of zinc dependence of magnetization can be ascribed as m = (μ_I_ + δμ`x) × μ_B_, where δ is delta equal to 1 if J_AB_ >> J_AA_ + J_BB_ and equal to −1 if J_AB_ << J_AA_ + J_BB_, μ_I_ is the initial magnetic moment of undoped ferrite and μ` is a concentration-depended magnetic moment resulting due to recombination of cations between lattices and disruption of ideal ferrimagnetic ordering. 

The linear fitting of region 0 < x < 0.25 gives the value (2.8 + 0.9x) × μ_B_. The value of magnetization per formula unit in the extremal case x = 0, pure cobalt ferrite, is very close to the theoretical value of 3 μ_B_ (only spin contribution to the magnetic moment it is taken into account) for a totally inverted spinel (γ = 1). In our case coefficient before x is much lower than estimated which indicates the Zn^2+^ occupy only partially tetrahedral sites while some amount of Zn^2+^ can occupy octahedral positions. This value is also affected due to antiferromagnetic order between magnetic cations in B sites since the B-B super-exchange interactions become more significant because of the breaking of the dominant A-B interactions. Spin canting arises when in B-sites concentration of non-magnetic ions becomes too high and can be explained in the frame of the Yafet-Kittel model [27,32,34]. Gómez-Polo and colleagues observed a divergence of the calculated value of magnetic moment by measured value of inversion degree by neutron diffraction which was enhanced when the concentration of zinc increased [34]. In our case trend becomes more pronounced in the region of 0.25 < x < 1 because of the change of the slope in the linear fitting of this region (4 − 3.6 x) × μ_B_. This non-monotonical trend of magnetization suggests that the filling of the spinel positions is also non-monotonical. The observed trend well agrees with literature data (adopted values from the literature of room temperature M_S_ are presented in Figure 3a). Mameli et al. observed maximal value M_S_ for ~7-nm particles prepared with thermal decomposition method for concentration of zinc of 0.46 at 5K, however at room temperature, the maxim of M_S_-x dependence shifted to the lower x values [25]. Ben Tahar et al. observed a maximum for x = 0.4 in ~5-nm particles prepared with the polyol method [32]. Andersen et al. found the maximum value of M_S_ at x = 0.2 for ~14-nm particles prepared with the hydrothermal method [51]. A small difference in the absolute value of magnetic moment and position of maximum related to the fact, that particles were prepared with different methods and have different sizes and cation distribution. 

The surface of spinel ferrites is preferentially formed by ions located in the octahedral sites. (Figure 3c). Thus, the chemical, catalytical and biological activity of mixed ferrites will mainly depend on structural properties (level of inversion degree) rather than on their chemical composition [38,39]. While the importance of the magnetic microstructure on the catalytical properties of nanoferrites is well known, their influence on biological activity is still relatively not well understood. 

The introduction of zinc in ferrite structure significantly decreases Curie (or Néel) temperature of ferrites MNPs which leads to the decrease of magnetization at room temperature. Previously it was demonstrated that the Curie temperature of cobalt ferrite MNPs was decreasing from 713 to 453 K when the concentration of zinc changed from x = 0 to 0.5 [52]. That makes this material potentially useful in, for example, the self-controlled hyperthermia [26,53] or any other applications where MNPs with high saturation magnetization and tunable Curie temperature are requested [54,55]. 

In contrast to this non-monotonically behavior of M_S_, the H_C_ trends to decreasing continuously as presented in Figure 2b as expected for the reduction of Co^2+^. It is interesting to underline the difference in comparison with a similar system of cobalt ferrite MNPs doped with nickel [37]. In the case of zinc, the H_C_ decreases much faster showing the downward trend, while for nickel-cobalt ferrite MNPs, the upward trend was observed. 

To better understand the role of magnetic anisotropy the constant of effective magnetic anisotropy (K_eff_) was calculated following [56]:

K_eff_ = M_S_·μ_0_H_A_/2, (4)

where μ_0_H_A_ is anisotropy field can be estimated by the closure field of hysteresis (H_irr_). This assumption was first suggested by Kodama et al. for NiFe_2_O_4_ MNPs [57] and it has been used for other similar nanoparticle systems [58,59]. Equation (4) is valid for non-interacting MNPs with uniaxial anisotropy. Despite, the magnetocrystalline anisotropy of cobalt ferrite is cubic type, in the case of nanoparticle system the effective anisotropy frequently turns on uniaxial type because of the contribution of such factors of shape, surface, and interparticle interactions [47]. K_eff_ calculated using equation 4 was maximal for CoFe_2_O_4_ 14 × 10^4^ J/m^3^ (the bulk 30 × 10^4^ J/m^3^ [60]), and fast drop until reaching a value in three-orders of magnitude lower for ZnFe_2_O_4_. The obtained M_R_/M_S_ and K_eff_ values are lower than expected probably due to the influence of temperature and interparticle interactions leading to the magnetization relaxation for particles with the lower anisotropy. Also, it is interesting to note a divergence in the behavior of coercivity and closure field, the H_irr_/H_C_ increased in two times for Zn-doped cobalt ferrites comparing with the pure cobalt ferrite MNPs. That confirms the crucial role of magnetocrystalline (core) anisotropy in cobalt ferrite samples and arising a stronger surface anisotropy in magnetically softer mixed Zn/Co ferrites MNPs.

### 3.3. Biology

Since the nanoparticles are iron-based, their presence should affect the bacteria iron metabolism system. Fe-containing MNPs may affect bacterial cells by reactive oxygen species production and induction of membrane disruption [61,62]. Thus, the data on the different effects of nanoparticles on wild-type cells and bacteria transformed with the plasmid can indirectly indicate the possibility of their penetration into the cell. The effect of iron ions on cell growth is a complicated parameter since iron is participating in many important biochemical processes, even if its overdose can cause a strong cytotoxic effect. To protect genomes from damage, *E.coli* uses the DNA-binding protein of starved cells (Dps), which combines ferroxidase activity and the ability to bind DNA. Both activities depend on the integrity of this multi-subunit protein, which has an inner cavity for iron oxides. It was shown by X-ray absorption near edge structure (XANES) and Mössbauer spectroscopy presence of both trivalent and divalent iron ions in the Dps protein [40,41]. Thus, if iron-based MNPs penetrate the cell, then they should affect the metabolic processes involving Dps.

**Figure 4:**
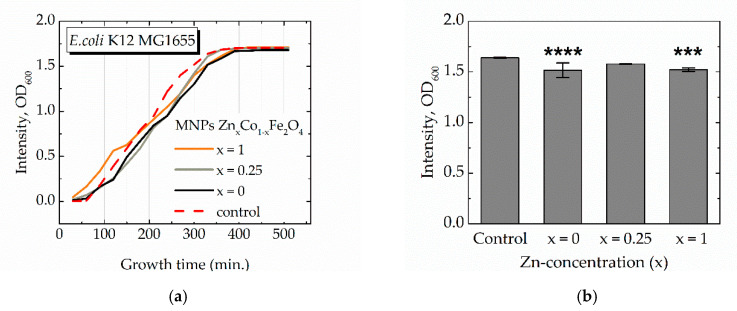
(**a**) Optical density (OD_600_) of *E.coli* K-12 MG1655 in LB medium as a function of cultivation time in presence of MNPs; (**b**) saturated value of OD_600_ after 330 min of cultivation (more data are in Figure S3).

Measured values of the OD_600_ signal are proportional to the cell’s population after the incubation time [63,64]. In the presence of CoFe_2_O_4_ and ZnFe_2_O_4_ MNPs, *E.coli* demonstrate a decreased OD_600_ of the bacterial medium compared with the control (non-treated) samples, resulting in the lag of bacterial growth inhibition in different timepoints (Figure 4a). The values of OD_600_ at 330 min, the time selected as moments when bacterial strains reached sub-maximum population, demonstrate that pure CoFe_2_O_4_ and ZnFe_2_O_4_ MNPs have a more pronounced lag effect than mixed Zn-Co ferrite MNPs with the concentration of zinc x = 0.25 (Figure 4b). In Figure 4, results for selected samples only are presented, more data confirming the same trend are in Figure S3. Cells growth of *E.coli* K-12 MG1655 was inhibited after MNPs (x = 1) from 240 to 330 min (*p*-value ≤ 0.001) and MNPs (x = 0) from 120 to 360 min (*p*-value ≤ 0.01). Therefore, it can be concluded that in the case of different Zn concentrations, a slowdown in the increase in OD is observed in the period from 200 to 400 min. Results cannot be attributed to the inhibition of bacterial growth that occurs during toxic exposure, since the achievement of the stationary growth phase occurs simultaneously for control cells and cells grown in the presence of nanoparticles. This bimodality can be explained by the triggering of special compensatory or protective mechanisms in bacterial cells. Such mechanisms can be implemented by post-translational modification [65], special chaperone proteins [66], or the triggering of special proteins of stresses [67].

The observed structural properties of the materials correlate with the non-monotonical effect of MNPs on bacterial growth. The toxicity of both Zn^2+^ and Co^2+^ ions, when they are incorporated in spinel structure, expected to be different, because of the occupation of different positions in spinel structure: octahedral positions are mostly located on the surface of particles and thus should have a stronger effect on biological and chemical properties. Nevertheless, zinc ferrite with Zn^2+^ mainly in tetrahedral positions and cobalt ferrite with Co^2+^ mainly in octahedral positions in our experiments showed similar behavior when bacteria were reached sub-maximum population. While the MNPs with the intermediate concentrations x = 0.25 do not exhibit a significant effect. This fact agrees with the non-monotonical evolution of the magnetic structure. Namely, at Zn concentration of around x = 0.25, Fe^3+^ ions partially migrate to octahedral positions lowering the toxic effect of Co^2+^ ions at the same positions. However, at high zinc concentration, Zn^2+^ ions start also occupy octahedral positions due to the non-thermodynamically equilibrium state of nanoparticles involving an increase of toxicity.

## 4. Conclusions

Zn-Co ferrite MNPs synthesized by the green synthesis method revealed tunable magnetic properties, which can be adjusted via their chemical composition. The increase of zinc content reduces magnetocrystalline anisotropy and has a non-monotonical effect on the saturation magnetization value with a maximal of ~74 Am_2_/kg for x = 0.25 zinc-concentration. This can be considered in a strong correlation with change of the magnetic structure of spinel structure: at small Zn-concentration (<25%), Zn^2+^ ions can occupy the tetrahedral sites pushing the Fe^3+^ with a high magnetic moment into the octahedral site. At a higher concentration of zinc, the ferrimagnetic order between two lattices is disturbed that leads to dropping in saturation magnetization and magnetic anisotropy. Here we should stress that magnetic properties correlate with microstructural and magnetic structural properties (first of all, cation distribution) which in their turn are in strong dependence on the synthesis method (in general, with the kinetic of reaction). Zn-Co ferrite MNPs induced the lag in *E.coli* growth, which was found in correlation with the magnetic and structural properties of spinel ferrites structure.

## Figures and Tables

**Table 1:** The crystallite size (D_XRD_), room temperature saturation magnetization (M_S_), coercivity field (μ_0_H_C_), reduced remanent magnetization (M_R_/M_S_) and effective magnetic anisotropy (K_eff_).

Sample Composition	D_XRD_, nm	M_S_,** Am^2^/kg	M_R_/M_S_, a.u.	μ_0_H_C_, mT	K_eff_, ×10^4^ J/m^3^
CoFe_2_O_4_	18(2) *	69(2)	0.42(2)	140(4)	14(2)
Zn_0.15_Co_0.85_Fe_2_O_4_	15(2)	71(2)	0.35(1)	76(3)	12(2)
Zn_0.25_Co_0.75_Fe_2_O_4_	16(2)	74(2)	0.34(1)	54(2)	9.5(4)
Zn_0.35_Co_0.65_Fe_2_O_4_	14(1)	65(2)	0.27(1)	37(2)	7.5(3)
Zn_0.50_Co_0.50_Fe_2_O_4_	15(2)	52(2)	0.22(1)	21(1)	3.3(1)
Zn_0.75_Co_0.25_Fe_2_O_4_	15(1)	32(1)	0.09(1)	5.5(2)	0.72(4)
ZnFe_2_O_4_	14(1)	10(1)	0.08(1)	4.8(2)	0.064(4)

* In parenthesis the systematical error in the last digit is presented; ** M_S_ values are after approximation with LAS, the error is fittings error.

## Supplementary Materials

The following are available online at https://www.mdpi.com/1996-1944/13/21/5014/s1, Figure S1. Antimicrobial susceptibility testing of E. coli, with experimental composition time 24h, using 6 mm filter-paper disks (a) containing Zn_25_Co_75_Fe_2_O_4_ MNPs and combination of different types of treatment (b). I-V - disks containing 16 mg/mL, 8 mg/mL, 4 mg/mL, 2 mg/mL, 1 mg/mL MNPs solution accordingly (5 μL of solution per disc), ab – disks containing 5 μL of antibiotic Kanamycin solution (50 μg/mL, used as positive control), E – empty segment, C - disks saturated with 5 μL distilled water, 1 – empty well used as a control, 2 – drop of 5 μL 16 mg/mL MNPs solution, 3 – well filled with 16 mg/mL MNPs solution, 4 – disk submerged in 16 mg/mL MNPs solution and placed on Lysogeny broth agar surface, 5 - 5 μL of 1 mg/mL MNPs solution covered by empty disk, 6 – disk saturated with 5 μL of 1 mg/mL MNPs. Red arrows show zones of inhibition surrounding disks containing antibiotics. Figure S2. Bright-field TEM image of CoFe_2_O_4_ nanoparticles prepared with sol-gel auto-combustion method: (a) low magnification and (b) higher magnification of the different areas of the same sample. Figure S3. (a) Optical density (OD600) of E.coli K-12 MG1655 in LB medium as a function of cultivation time in presence of MNPs; (b) saturated value of OD600 after 330 min of cultivation.
