# Supplementary material for: Green Synthesis of Co-Zn Spinel Ferrite Nanoparticles: Magnetic and Intrinsic Antimicrobial Properties
Source: Materials (Basel). 2020 Nov 6;13(21):5014. doi: 10.3390/ma13215014 (PMC7664412; doi:10.3390/ma13215014)
Supplement: Supplementary file 1 [file materials-13-05014-s001.pdf]

materials

MDPI

Supplementary materials

Green Synthesis of Co-Zn Spinel Ferrite Nanoparticles: Magnetic and Intrinsic Antimicrobial Properties

Alexander Omelyanchik [1], Kateryna Levada [1], Stanislav Pshenichnikov [1], Maryam Abdolrahim [2], Miran Baricic [3], Anastasiya Kapitunova [4], Alima Galieva [4], Stanislav Sukhikh [4], Lidiia Astakhova [4], Sergey Antipov [4,5], Bruno Fabiano [6], Davide Peddis [2,3,*] and Valeria Rodionova [1,*]

[1] Institute of Physics, Mathematics and Information Technology, Immanuel Kant Baltic Federal University, 236041 Kaliningrad, Russia; asomelyanchik@kantiana.ru (A.O.); elevada@kantiana.ru (K.L.); spshenichnikov1@kantiana.ru (S.P.)

[2] Institute of Structure of Matter–CNR, 00016, Monterotondo Stazione, 00015 Rome, Italy; mabdolrahimi@os.uniroma3.it (M.A.)

[3] Department of Chemistry and Industrial Chemistry (DCIC), University of Genova, 16146 Genova, Italy; S4193366@studenti.unige.it (M.B.)

[4] School of Life Science Immanuel Kant Baltic Federal University, 236041 Kaliningrad, Russia; aikapitunova@stud.kantiana.ru (A.K.); agalieva@stud.kantiana.ru (A.G.); ssukhikh@kantiana.ru (S.S.); lastakhova@kantiana.ru (L.A.); santipov@kantiana.ru (S.A.)

[5] Research Center "Molecular basis of Biotechnology of Living Systems", K.G. Razumovsky Moscow State University of Technologies and Management (the First Cossack University), 109004 Moscow, Russia

[6] Department of Civil, Chemical and Environmental Engineering, Polytechnic School, University of Genova, Via Opera Pia 15, 16145 Genova, Italy; brown@unige.it (B.F.)

* Correspondence: davide.peddis@unige.it (D.P.) and vvrodionova@kantiana.ru (V.R.)

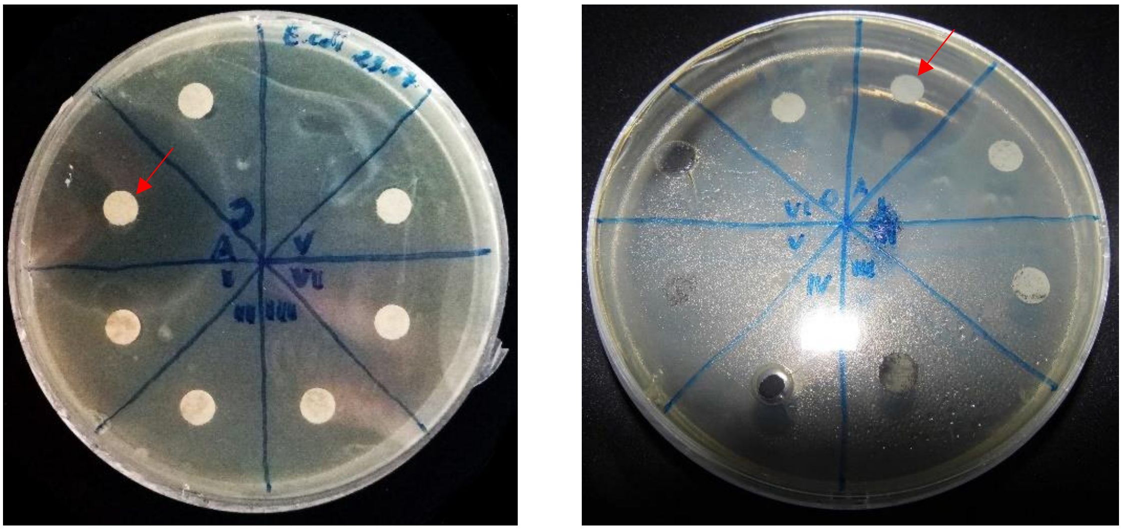

(**a**) (**b**)

Figure S1. Antimicrobial susceptibility testing of *E. coli*, with experimental composition time 24h, using 6 mm filter-paper disks **(a)** containing $Zn_{25}Co_{75}Fe_2O_4$ MNPs and combination of different types of treatment **(b)**. I-V - disks containing 16 mg/ml, 8 mg/ml, 4 mg/ml, 2 mg/ml, 1 mg/ml MNPs solution accordingly (5 µl of solution per disc), ab – disks containing 5 µl of antibiotic Kanamycin solution (50 µg/ml, used as positive control), E – empty segment, C - disks saturated with 5 µl distilled water, 1 – empty well used as a control, 2 – drop of 5 µl 16 mg/ml MNPs solution, 3 – well filled with 16 mg/ml MNPs solution, 4 – disk submerged in 16 mg/ml MNPs solution and placed on Lysogeny broth agar surface, 5 - 5 µl of 1 mg/ml MNPs solution covered by empty disk, 6 – disk saturated with 5 µl of 1 mg/ml MNPs. Red arrows show zones of inhibition surrounding disks containing antibiotics.

To evaluate MNPs antimicrobial effects disk diffusion method was performed. Lysogeny broth (LB) agar medium was homogeneously distributed on Petri dishes (PD). After medium solidification bacterial cells were evenly distributed on medium surface using glass spreaders. Filter-paper disks (6 mm in diameter) were saturated with MNPs solutions (with 1–16 mg/ml concentrations) and placed on LB surface. Additionally, other types of MNPs placement on LB surface were used (wells on LB agar were filled with MNPs solution). In the next step cells were incubated aerobically at 37 °C for 24h. Later zones of bacterial inhibition growth were analyzed. Images of antimicrobial susceptibility testing were evaluated using ImageJ software [1].

Experiments based on the disk diffusion method demonstrated the absence of bacterial inhibition effects on LB surface after 24h MNPs treatment - only disks saturated with antibiotic Kanamycin (50 µg/ml solution, used as positive control) showed zones of *E.coli* inhibition (Figure 1Sa). Additionally, other types of MNPs treatment based on the disk diffusion method and his variations illustrated in (Figure 1Sb) demonstrated no inhibition effects for all tested MNPs.

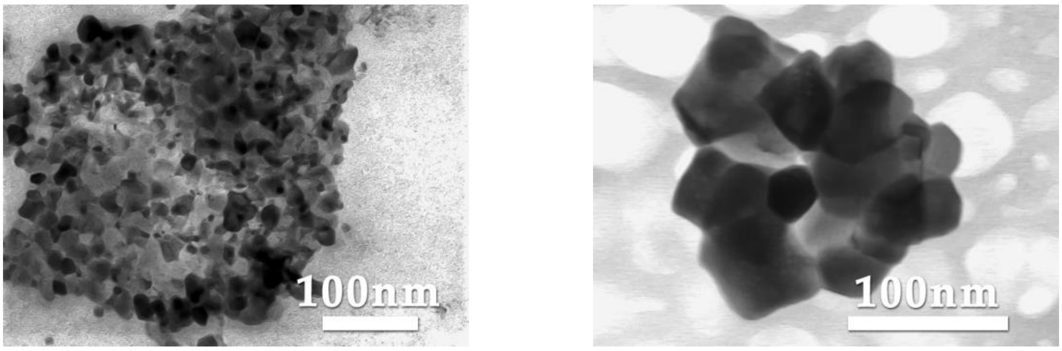

(**a**) (**b**)

Figure S2. Bright-field TEM image of $CoFe_2O_4$ nanoparticles prepared with sol-gel auto-combustion method: (**a**) low magnification and (**b**) higher magnification of the different areas of the same sample.

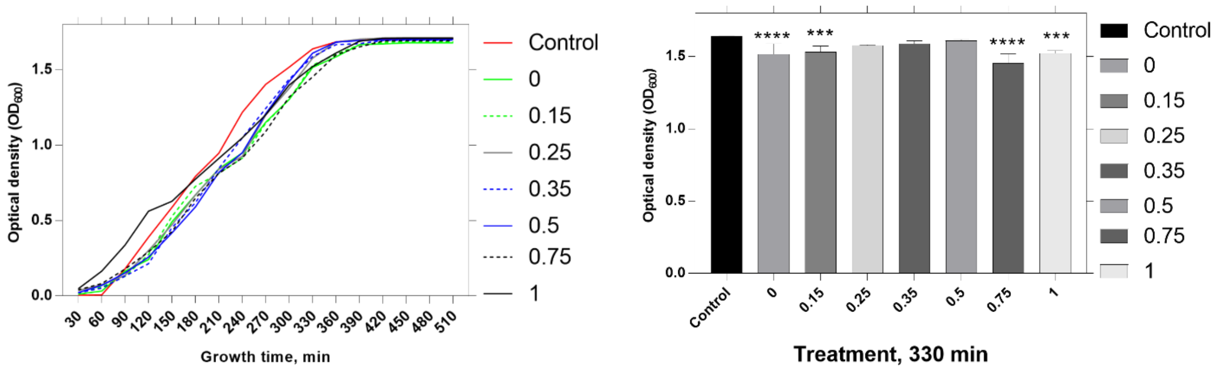

(**a**) (**b**)

Figure S3. (**a**) Optical density (OD_{600}) of *E.coli* K-12 MG1655 in LB medium as a function of cultivation time in presence of MNPs; **(b)** saturated value of OD_{600} after 330 min of cultivation.

References:

[1] Schneider, C.A.; Rasband, W.S.; Eliceiri, K.W. NIH Image to ImageJ: 25 years of image analysis. *Nat. Methods* **2012**, *9*, 671–5.
